# Synthesis of Phenolics and Flavonoids in Ginger (*Zingiber officinale* Roscoe) and Their Effects on Photosynthesis Rate

**DOI:** 10.3390/ijms11114539

**Published:** 2010-11-15

**Authors:** Ali Ghasemzadeh, Hawa Z. E. Jaafar, Asmah Rahmat

**Affiliations:** 1 Department of Crop Science, Faculty of Agriculture, University Putra Malaysia, 43400 UPM Serdang, Selangor, Malaysia; E-Mail: upmali@yahoo.com; 2 Department of Nutrition & Dietetics, Faculty of Medicine & Health Sciences, University Putra Malaysia, 43400 UPM Serdang, Selangor, Malaysia; E-Mail: asmah@medic.upm.edu.my

**Keywords:** photosynthesis, flavonoids, salicylic acid, Halia Bentong, Halia Bara

## Abstract

The relationship between phenolics and flavonoids synthesis/accumulation and photosynthesis rate was investigated for two Malaysian ginger (*Zingiber officinale*) varieties grown under four levels of glasshouse light intensity, namely 310, 460, 630 and 790 μmol m^−2^s^−1^. High performance liquid chromatography (HPLC) was employed to identify and quantify the polyphenolic components. The results of HPLC analysis indicated that synthesis and partitioning of quercetin, rutin, catechin, epicatechin and naringenin were high in plants grown under 310 μmol m^−2^s^−1^. The average value of flavonoids synthesis in leaves for both varieties increased (Halia Bentong 26.1%; Halia Bara 19.5%) when light intensity decreased. Photosynthetic rate and plant biomass increased in both varieties with increasing light intensity. More specifically, a high photosynthesis rate (12.25 μmol CO_2_ m^−2^s^−1^ in Halia Bara) and plant biomass (79.47 g in Halia Bentong) were observed at 790 μmol m^−2^s^−1^. Furthermore, plants with the lowest rate of photosynthesis had highest flavonoids content. Previous studies have shown that quercetin inhibits and salicylic acid induces the electron transport rate in photosynthesis photosystems. In the current study, quercetin was an abundant flavonoid in both ginger varieties. Moreover, higher concentration of quercetin (1.12 mg/g dry weight) was found in Halia Bara leaves grown under 310 μmol m^−2^s^−1^ with a low photosynthesis rate. Furthermore, a high content of salicylic acid (0.673 mg/g dry weight) was detected in Halia Bara leaves exposed under 790 μmol m^−2^s^−1^ with a high photosynthesis rate. No salicylic acid was detected in gingers grown under 310 μmol m^−2^s^−1^. Ginger is a semi-shade loving plant that does not require high light intensity for photosynthesis. Different photosynthesis rates at different light intensities may be related to the absence or presence of some flavonoid and phenolic compounds.

## 1. Introduction

Ginger is an important horticultural crop in tropical Southeast Asia. It produces a pungent aromatic rhizome that is valuable all over the world as either a spice or herbal medicine [1]. In Malaysia, it has been used as a food and medicinal plant for over 2000 years for treating diabetes, high blood pressure, cancer and many other illnesses [1]. In recent studies, ginger varieties have been reported as a good potential source for anti-cancer, anti-microbial and anti-inflammation [2]. The bioactive molecules of ginger are 6-gingerol, flavonoids and phenolic acids [3]. Quality control of active ingredients in herbs, safety and environmental conservation has recently become serious issues in herb production. The concentration of secondary metabolites of plants can also be influenced by environmental conditions such as light intensity, temperature, insects, biotic and abiotic factors, which can alter the concentration of the active constituents and can be harmful to consumers [4,5]. Southwell *et al*. [4] have reported that the contents of some medicinal compounds (hypericin and pseudohypericin) in the leaves of *Hypericum perforatum* varied up to 50-fold between summer and winter grown plants. Therefore, growing plants under a controlled environment can be considered an alternative way to ensure safety and efficiency. When plants are grown under a controlled environment with simulated lighting, it is possible to enhance plant yield production with minimum use of resources and minimum or no pollutants released to the environment [6]. Under a controlled environment, uniform growth of plants can be expected and production planning and scheduling can be possible, and contamination by unexpected factors such as diseases, insects, metals and other harmful factors can be reduced or eliminated [6]. Recently, researchers have reported that some environmental factors such as light intensity and CO_2_ concentration can significantly alter the secondary metabolite synthesis and production in plants. Light is known to adjust not only plant growth and development, but also the biosynthesis of primary and secondary metabolites [7,8]. The synthesis of medicinal components in herbs is affected by light intensity with changes in plant morphology and physiology characteristics [7,9,10].

Briskin *et al.* [10] concluded that hypericin synthesis increased significantly in *H. perforatum* when grown under high light intensity (400 μmol m^−2^s^−1^). It seems that a high photosynthetic rate under high light intensity resulted in an increased amount of carbon assimilation and enhanced the secondary metabolites in the leaf tissues. In contrast, some researchers have obtained a high rate of secondary metabolite synthesis and content in non-photosynthetic tissues by enhancing the light intensity. Kurata *et al.* [7] reported that the high light irradiation enhanced purine alkaloid (caffeine and theobromine) content in *Coffea arabica* due to the physiological changes in cell growth. Zhong *et al.* [8] found that anthocyanin production increases in the cell culture of *Perilla frutescens* (shiso) with increasing light intensity. Phenolic biosynthesis requires light or is enhanced by light, whereas flavonoids formation is absolutely light-dependent, and its biosynthetic rate is related to light intensity and density [11]. Previous studies showed that changes in light intensity are capable of changing the production of flavonoids and phenolics in herbs [12]. Furthermore, Chan *et al.* [13] reported much greater/higher concentration of flavones and flavonols in the leaves of vegetables that are exposed to shade. This finding is in agreement with Bergquist *et al.* [14], who indicated that the use of shade netting is acceptable for the production of baby spinach in relation to flavonoid concentration and composition. A similar trend of increasing total flavonoids (TF) content with decreasing light intensity was seen in *Tanacetum parthenium* and strawberry [15,16] and in some medicinal plants, illustrating considerable influence of low irradiance on enhancement of plant TF [9]. Michel *et al.* [17] reported TF production related to plant pigments (chlorophyll and carotenoids). In contrast with flavonoids, the xanthophyll cycle seems to be mainly relevant to the protection of photosynthesis against sudden increase in light intensity. Concurrently, it is necessary to consider whether the increased amount of secondary metabolites obtained under different light intensity is due to the increased amount of carbon production through photosynthesis or the stress induced by different light intensities, which stimulates secondary metabolites production. Flavonoids are important in plant biochemistry and play an important role in plant physiology, acting as antioxidants, enzyme inhibitors, pigments and light screens. These compounds are involved in photosensitization and electron transfer, growth regulation, photosynthesis and defense against infection [18]. It is possible that a relationship exists between flavonoids production and photosynthesis rate in plants. Sergio *et al.* [19] found that the first step in plant photosynthesis could be repressed by flavonoids and/or shikimic acid, with subsequent shift of carbon flux into secondary metabolism. He reported that under conditions of excess products of glycolysis (including PEP), or photosynthetic metabolites, the synthesis of secondary aromatic products increases. Though previous studies on the relationship between photosynthesis and flavonoids content have been carried out, most of the results remain contradictory. There are three different viewpoints: (1) There is a notable positive relationship between flavonoid content and photosynthesis [11]; (2) there is a positive but indirect relationship under given conditions. For example, the enhancement of carbohydrates resulted in an increase of flavonoids content in plant tissue culture [20]; (3) there is no relationship, not even a negative one.

The objectives of this study were to consider the effect of different natural light intensities on photosynthesis rate, primary (soluble carbohydrates) and secondary metabolites synthesis (flavonoids and phenolics acids) in Malaysian ginger varieties and to determine any relationship between photosynthesis rate and flavonoids and phenolics synthesis in these conditions.

## 2. Results and Discussion

### 2.1. Flavonoids and Phenolics Content (HPLC Analysis)

High performance liquid chromatography (HPLC) analysis of flavonoids is present in Table 1. According to the data obtained (Table 1), the concentration of the majority flavonoids (quercetin, rutin, catechin, epicatechin and naringenin) was increased in plants when grown under 310 μmol m^−2^s^−1^. Accumulation of the studied flavonoid components from the sink (leaves) to the source (rhizomes) increased under low light intensity. The analysis of flavonoid components using HPLC in leaves of ginger showed that quercetin possessed the highest concentration, followed by cathechin. The analysis of quercetin concentration under two light intensities indicated a higher concentration of quercetin in ginger leaves grown under 310 μmol m^−2^s^−1^ compared with plants grown under 790 μmol m^−2^s^−1^. In addition, quercetin decreased the photosynthesis rate through inhibition of the ATPase activity and electron transport rate in photosynthesis photosystems [21].

Rutin in Malaysian ginger ranged from 0.173 and 0.451 mg/g dry weight. The highest concentration of rutin was recorded in rhizome of Halia Bentong exposed to low light; however, when light intensity increased, accumulation of rutin in rhizome decreased by about 31%. Rutin concentration in Halia Bentong leaves was low, especially when exposed to high light intensity. A moderate concentration of rutin was observed in Halia Bara rhizome under a high light level (0.324 mg/g dry weight). Rutin accumulation in Halia Bara, although still favored in the rhizomes more than the leaves, declined in concentration in both plant parts with increasing light intensity from 310 to 790 μmol m^−2^s^−1^.

Catechin and epicatechin are polyphenolic antioxidant plant secondary metabolites. The term catechin is also commonly used to refer to the related family of flavonoids and the subgroup flavanols. Catechin concentration in Halia Bara under low light intensity was higher in the leaves than rhizomes, which was also comparable to that contained in the rhizomes of Halia Bentong under low light. Meanwhile, epicatechin concentration was highest in leaves of both varieties under low light intensity (0.117–0.118 mg/g dry weight) although the amount was not clearly different from those obtained in the rhizome of Halia Bara under low or high light conditions. Generally, the concentration of epicatechin in Halia Bentong was lower than that found in Halia Bara, especially in rhizomes under high light intensity (0.078 mg/g dry weight).

Kaempferol is a rare flavonoid in plants. However, in the leaves and rhizomes of Halia Bara and Halia Bentong, it was detected in small concentrations (between 0.042 and 0.068 mg/g dry weight).

Naringenin is a flavonoid that is considered to have a bioactive effect on human health as an antioxidant, free radical scavenger, anti-inflammatory, carbohydrate metabolism promoter, and immune system modulator. It is the predominant flavanone in grapefruit and was found to have an inhibitory effect on carcinogens [22]. Although a lack of information has been gathered about naringenin in ginger, from the present study, its concentration was low, ranging from 0.02 to 0.094 mg/g dry weight. Naringenin concentration in ginger was clearly affected by the differences in varieties, light intensity, and plant parts. Generally, Halia Bentong had a higher concentration than Halia Bara, with more accumulation found in the leaves than in the rhizomes, especially under low light condition.

Irradiance increases leaf area-based phenolics content, which is mainly accumulated in the epidermis [23,24]. Shui *et al.* [25] found that ecological factors influenced flavonoid concentration primarily during the young stage of *Ginkgo biloba* development; and amongst the ecological factors studied, light and temperature had the greatest effects on flavonoid synthesis in *Ginkgo biloba*.

Salicylic acid, belonging to plant phenolics group, is found in some plant species, and its highest levels are observed in the inflorescence of thermogenic plants and in spice herbs [26]. According to Table 1, salicylic acid was not detected in gingers grown under a light intensity of 310 μmol m^−2^s^−1^. A high content of salicylic acid (0.673 mg/g dry weight) was detected from Halia Bara leaf extract grown under 710 μmol m^−2^s^−1^ light intensity. The results of previous studies showed that salicylic acid is capable of enhancing plant growth and yield. Jeyakumar *et al.* [27] reported that salicylic acid was able to enhance the dry matter production in blackgram. Induction of photosynthesis rate and stomatal conductance by salicylic acid was provided in previous studies [28].

Nevertheless, although the leaves’ flavonoid content is highly sensitive to biotic and abiotic control of PAL expression [29], the results of Waterman *et al.* [30] and Mole *et al.* [31] showed parallel variations of phenolics and flavonoids under different irradiance levels. Contrary to our results, higher phenolics content was reported in the rhizomes of *Z. officinale* rather than its leaves [32]. In addition, the results of Chan *et al.* [13], reporting a high level of flavonoid components in ginger leaves compared to the rhizomes of *Z. officinale*, supported our finding. The synthesis of isoflavones and some other flavonoids is induced when plants are infected or injured [33,34], or under low light and low nutrient condition [22,34]. The increase in soluble phenolics such as intermediates in lignin biosynthesis can reflect the typical anatomical change induced by stressors: An increase in cell wall endurance and the creation of physical barriers prevent walls against harmful actions [35]. However, some plant products such as anthocyanin, cumarin and lignin are biosynthesized while phenolic compounds are being transformed into flavonoids [36]. From Table 2, it can be observed that Halia Bentong had a higher average increase in flavonoid components (26.1%) compared to Halia Bentong (19.5%) when the light intensity was decreased.

From Table 1, it is apparent that total flavonoids (TF) and total phenolics (TP) accumulation and partitioning in the plant were significantly affected by the differing light intensities (*p* ≤ 0.01). With decreasing TP in the leaves and rhizomes observed when decreasing light intensity from 790 to 310 μmol m^−2^s^−1^, TF content increased significantly in the leaves and rhizome of both varieties.

In this study, cinnamic acid was not detected in plants grown under 310 μmol m^−2^s^−1^ where instead high content of flavonoids was registered; but cinnamic acid was detected in ginger grown under 790 μmol m^−2^s^−1^ with low content of flavonoids.

These results suggest the ability of different light intensities to alter or modify both the concentration and profiling of phenolic components in ginger plants; although accumulation of phenolics components favored high light intensity, in contrast, low light intensity generally promoted the accumulation of flavonoids. Considering the intricacy of flavonoid biosynthesis and flavonoid metabolism processes, it is difficult to figure out linear relationship between flavonoids and their basic precursors.

### 2.2. Photosynthesis Rate, Stomata Conductance and Transpiration

The leaf net photosynthetic rate, stomata conductance and transpiration rate increased with increasing light intensity (Table 3). According to the results in Table 3, a high photosynthesis rate (12.25 μmol CO_2_ m^−2^s^−1^) was obtained in Halia Bara varieties when grown under 790 μmol m^−2^s^−1^. Under low light intensity (310 μmol m^−2^s^−1^), the stomata conductance was low compared to plants grown under high light intensity (790 μmol m^−2^s^−1^). Stomata behavior and regulation are very important factors in the control of photosynthetic rate. The current study also showed that increasing light intensity from 310 to 790 μmol m^−2^s^−1^ increased the net photosynthetic rate significantly as well as the stomata conductance and transpiration rate. This suggests that the increase in photosynthetic rates resulted from increased CO_2_ uptake activity at the chloroplast level, rather than simple increases in stomata opening (reduced resistance to CO_2_ entry in the leaves). Either situation could lead to an increase in photosynthetic rate, however, when an increase in stomata opening is the primary cause of increased photosynthetic activity, an increase in internal carbon would be expected. Ajithkumar *et al*. [37] point out that photosynthetic rate, stomata conductance, transpiration rate, stomatal index, and stomatal frequency decreased significantly with increasing shade level. The results of the current study showed that increasing flavonoids in ginger decreased the photosynthesis rate significantly and *vice versa*. A possible explanation is that flavonoids and shikimic acid could be part of the regulatory system controlling the flux of carbon into secondary compounds in plants [38]. Consequently, decreasing the photosynthesis rate resulted in increased shikimic acid activity and flavonoid contents. The effect of shikimic acid inhibition on photosynthesis enzymes is supported by Dixon plots study [39]. Further, competitive inhibition pattern on PEP carboxylase activities was also reported previously [39]. Although previous studies on the relationship between photosynthesis and flavonoids content have been carried out, most of the results remain contradictory. A number of studies have found that increased photosynthesis and carbohydrate content in leaves and dry weight of leaves were not accompanied by increased flavonoid content and its synthesis speed [39]. Stomata conductance, photosynthesis rate and transpiration also increased with increasing phenolic compounds [40]. The mechanism involved in increased photosynthetic rates and leaf area is not known.

When photosynthesis occurs with the presence of light, flavonoid components are able to change the rate of electron transport and photophosphorylation, bringing about the change of ATP/NADPH ratio [21]. In carbon metabolism reactions, they can shift the dynamic equilibrium of pentosephosphate reduction cycle to enhance the synthesis of main metabolites due to both the change in energy substrate intake and the interaction with enzymes of the cycle. Additionally, flavonoids exercise a feedback control over their own biosynthesis, although this phenomenon is not clearly understood [41]. However, inhibitory effect of flavonoids on photosynthesis rate was reported in previous studies [21]. As opposed to the shade leaves, the sun leaves typically exhibit high photosynthetic capacity [42], therefore, they have high carbon input, which can exceed the demand for protein synthesis and stimulate phenolic synthesis [43].

Ginger is a semi-shade loving plant that does not require high light intensity for its photosynthesis [44]. Furthermore, the difference in photosynthesis rate under different light intensities may be related to the absence or presence of salicylic acid in ginger. Hence, further study is required to establish the effect of salicylic acid on photosynthesis rate in ginger.

### 2.3. Chlorophyll Content

Chlorophyll concentration significantly increased with decreasing light intensity from 790 to 310 μmol m^−2^s^−1^. A high concentration of chlorophyll was obtained in Halia Bara 273.5 μg mL^−1^ grown under 310 μmol m^−2^s^−1^. Such an increase in chlorophyll content when grown under shade conditions was reported by Khan *et al.* [45] and Souza *et al.* [46]. Chl a:b ratio decreased with increasing light intensity (Table 3). With increasing chlorophyll a + b content, TF increased but TP decreased in both varieties. This negative relationship between TP and chlorophyll a + b content was not significant. Competition between TP and chlorophyll synthesis was reported in previous studies [46,47]. The described competition between chlorophyll and phenolics in leaves fits well with the predictions of the Protein Competition Model (PCM), that is, that the total leaf mass-based polyphenols content is controlled by the competition between protein and polyphenol biosynthetic pathways and its metabolic regulation. This indicates an accumulation of dry matter that dilutes chlorophyll and polyphenols [47]. The increased TF in Halia Bentong with increasing shading could be associated with significantly higher leaf chlorophyll and carotenoids contents under lower light levels. Michel *et al.* [17] concluded that TF production is related to plant pigments (chlorophyll and carotenoids), and in contrast to flavonoids, the xanthophyll cycle seem to be mainly relevant to protect photosynthesis against sudden increases in light intensity. Increasing chlorophyll content is usually followed by an increase in flavonoid content in plant [47].

### 2.4. Total Soluble Carbohydrate (TSC)

Different light intensities significantly affected TSC content (Table 1). With increasing light intensity, TSC was increased significantly (*p* ≤ 0.01) in both varieties. Halia Bara had a higher content of TSC (18.49 mg/g dry weight) compared with Halia Bentong (17.4 mg/g dry weight) when the varieties were exposed to 790 μmol m^−2^s^−1^ light intensity. A positive and significant (*p* ≤ 0.01) correlation was observed between photosynthesis and TSC content. In addition, correlation between TP synthesis and TSC was positive and significant but the correlation between TF and TSC was not significant. On the contrary, flavonoid accumulation was observed when carbohydrate (sugar) content was lower [42]. The shikimic acid pathway participates in the biosynthesis of most plant phenolics. Soluble carbohydrates are basic compounds required to produce phenolic component in the shikimic acid pathway: The shikimic acid pathway is able to convert simple carbohydrate precursors derived from glycolysis and the pentose phosphate pathway to the aromatic amino acids [43]. The results of previous studies showed an increase in phenolic concentration related to the balance between carbohydrate source-sink, such that the greater the source:sink ratio, the greater the concentration of phenolic compounds [25,43]. In other words, high light intensity (790 μmol m^−2^s^−1^) enhanced soluble carbohydrate content through increasing the photosynthesis rate, thereby phenolics synthesis in ginger varieties were enhanced.

A positive and highly significant correlation between total phenolics and soluble carbohydrate was observed in this study (Table 4). It was also observed that ginger with a high content of salicylic acid had higher soluble carbohydrate content. These results are in agreement with Amin *et al.* [48], who reported that salicylic acid regulates sugar contents (translocation from source to sink) and causes a significant increase in total soluble sugars.

### 2.5. Plant Biomass

Different light intensities significantly affected (*p* ≤ 0.001) plant biomass production. With increasing light intensity, plant biomass increased significantly. A high content of plant biomass (75.6 g) was observed in Halia Bentong grown under a light intensity of 790 μmol m^−2^s^−1^. The quality and yield of ginger rhizomes increases when grown in the shade due to increased nutrient uptake [32].

According to previous studies, high ginger biomass was obtained when grown under a light intensity of 800 μmol m^−2^s^−1^ [49]. Increasing TSC in plants grown under 790 μmol m^−2^s^−1^ of light intensity was one of the parameters that could enhance plant biomass. According to our results, with an increased photosynthesis rate, biosynthesis of TP increased in ginger varieties, and previous studies showed that some phenolic compounds such as salicylic acid are able to regulate and enhance plant growth. A recent study by Nagasubramaniam *et al.* [26] showed that salicylic acid (100 ppm) increased the plant height, leaf area, crop growth rate and total dry matter production in baby corn. Jeyakumar *et al.* [27] reported that salicylic acid (125 ppm) was able to enhance the dry matter production in blackgram. A positive correlation was found between plant biomass and photosynthesis rate (Table 4).

## 3. Experimental

### 3.1. Plant Material and Maintenance

Rhizomes of ginger varieties, Halia Bentong and Halia Bara (*Zingiber officinale*), were germinated for two weeks in a small pots. They were then transferred to white polyethylene bags, which were filled with soilless mixture media including burnt rice husk and coco peat (ratio 1:1) The plants were grown under four levels of glasshouse shade (0%, 20%, 40% and 60% shade) at the glasshouse complex of University Putra Malaysia (UPM). The average light intensity passing through to each shading treatment was 790, 630, 460 and 310 μmol m^−2^s^−1^ of photosynthetically active radiation (PAR), respectively. Relative humidity was 70 ± 5% and average temperature was 29 °C. The experiment was factorial based on Randomized Complete Block with three replications. The experiment was conducted for 16 weeks. Plants were harvested and fresh and dry mass of leaves, stems and rhizomes and number of stem nodes was measured.

### 3.2. Extract Preparation for TP and TF Measurement

Leaves, stems and rhizomes were freeze dried to constant weights before using in the extraction. For antioxidant analysis, the leaves, stems and rhizomes were made into powder and one gram of the powder was used in the extraction, using methanol (50 mL). The solutions were shaken for 1 h at room temperature using an orbital shaker. Extracts were filtered under suction and stored at −20 °C until further use.

### 3.3. Determination of Total Phenolics Contents

The total phenolics content was determined using Folin-Ciocalteu reagents with analytical grade gallic acid as the standard. 1 mL of extract or standard solution (0–500 mg/L) was added to deionized water (10 mL) and Folin-Ciocalteu phenol reagents (1.0 mL). After 5 minutes, 20% sodium carbonate (2.0 mL) was added to the mixture. After being kept in total darkness for 1 h, the absorbance was measured at 750 nm using a spectrophotometer (U-2001, Hitachi Instruments Inc., Tokyo, Japan). Amounts of TP were calculated using a gallic acid calibration curve. The results were expressed as gallic acid equivalents (GAE) g/g of dry plant matter [49].

### 3.4. Determination of Flavonoids Contents

Total flavonoids content was measured following the method of Bushra *et al.* [50]. Briefly, extracts of each plant material (1 mL containing 0.1 mg/mL) were diluted with water (4 mL) in a 10 mL volumetric flask. Initially, 5% NaNO_2_ solution (0.3 mL) was added to each volumetric flask; at 5 min, 10% AlCl_3_ (w/w) was added; at 6 min, 1.0 M NaOH (2 mL) was added. Water (2.4 mL) was then added to the reaction flask and mixed well. Absorbance of the reaction mixture was read at 430 nm. The results were expressed in mg quercetin/g dry weight by comparison with the quercetin standard curve.

### 3.5. High Performance Liquid Chromatography (HPLC) Apparatus

#### 3.5.1. Extract Preparation

0.25 g aliquots of leaves and rhizomes were extracted with 20 mL 60% aqueous methanol. 5 mL 6 M HC1 was added to each extract to give a 25 mL solution of 1.2 M HC1 in 50% aqueous methanol. Extracts were refluxed at 90 °C for 2 h. Extract aliquots of 500 μL, taken both before and after hydrolysis, were filtered through a 0.45 μm filter [51].

#### 3.5.2. HPLC Analysis of Flavonoids

Reversed-phase HPLC was used to assay the composition of flavonoids. Agilent HPLC system (Tokyo, Japan) consisted of a Model 1100 pump equipped with a multi-solvent delivery system and L-7400 ultraviolet (UV) detector. The column type was Agilent C18 5 μm, 4 mm internal diameter 250 mm. The mobile phase was composed of (A) 2% acetic acid (aqueous) and (B) 0.5% acetic acid (aqueous)-acetonitrile (50:50 v/v), and gradient elution was performed as follows: 0 min, 95:5; 10 min, 90:10; 40 min, 60:40; 55 min, 45:55; 60 min, 20:80; and 65 min, 0:100. The mobile phase was filtered under vacuum through a 0.45 μm membrane filter before use. The flow rate was 1 mL/min. UV absorbance was measured at 280 nm and, for flavone, at 365 nm. The operating temperature was maintained at room temperature [52]. Identification of the flavonoids was achieved by comparison with retention times of standards, UV spectra and calculation of UV absorbance ratios after co-injection of samples and standards. Commercial standards were purchased from Sigma-Aldrich (USA).

#### 3.5.3. Extract Preparation of Phenolics

1.2 mL phosphoric acid (H_3_PO_4_) was dissolved into about 950 mL water in a 1 L volumetric flask and brought to volume with water. 0.25 g of leaves and rhizomes were extracted with 20 mL of phosphoric acid. 5 mL 6 M HC1 was added to each extract to give a 25 mL solution of 1.2 M HC1 in 50% aqueous methanol. Extracts were refluxed at 90 °C for 2 h and solutions were filtered through a 0.45 μm filter [53].

#### 3.5.4. HPLC Analysis of Phenolics

Agilent HPLC system (Tokyo, Japan) consisted of a Model 1100 pump equipped with a multi-solvent delivery system and a L-7400 ultraviolet (UV) detector. The column type was Agilent C18, 5 μm, 4.6 mm internal diameter 250 mm. The mobile phase was composed of phosphoric acid (aqueous) and (B) acetonitrile gradient elution was performed as follows: 0 min, 85:15; 12 min, 75:25; 20 min, 75:25; 22 min, 85:15; and 30 min, 85:15. The mobile phase was filtered under vacuum through a 0.45 μm membrane filter before use. The flow rate was 1 mL/min and injection volume was 20 μL. UV absorbance was measured at 220–365 nm. The operating temperature was maintained at room temperature [53]. Identification of the flavonoids was achieved by comparison with retention times of standards, UV spectra and calculation of UV absorbance ratios after co-injection of samples and standards. Commercial standards were purchased from Sigma-Aldrich (USA).

### 3.6. Photosynthesis Rate, Stomata Conductance, Transpiration

Photosynthetic rate, stomata conductance and transpiration rate of fully expanded leaves was measured by using a portable photosynthesis system (LICOR-64001 LI-COR Inc., USA).

### 3.7. Chlorophyll Measurement

For the measurement of chlorophyll concentration, the weighed fresh leaves (200 mg) were grinded using a mortar and pestle and immersed in 10 mL of 100% acetone. Samples were wrapped in aluminum foil and homogenized with the B-Brawn type homogenizer at 1000 rpm for one minute. The homogenate was filtered through two layer of cheesecloth, and was centrifuged at 2500 rpm for 10 minutes. The supernatant was separated and placed in quartz cuvettes and absorbance measured against a blank of 100% acetone at 2 wavelengths. The two wavelengths of 662 nm and 645 nm were used as the peak absorbences of chlorophyll-a and chlorophyll-b. The total amount of chlorophyll-a and chlorophyll-b were then calculated according to the formulas of Lichtentaler and Wellburn [54].

### 3.8. Determination of Total Soluble Carbohydrate (TSC)

A few drops of ethanol (80%) were added to 0.1 g of dry samples and then 25 mL aqueous ethanol (5 mL water + 20 mL 80% ethanol) was added and mixed via shaking. Solutions were centrifuged at 5000 rpm, the supernatant was separated and filtrated. Filtrate volume was adjusted to 100 mL with water. Approximately 1 mL of supernatant was placed into test tubes, 10 mL of anthron solution (0.15%) was added, and finally the samples were heated in a water bath at 95 °C for 8 min. Tubes were immediately transferred into an ice bath and cooled down to room temperature. Absorption of the samples was recorded at 625 nm [55].

### 3.9. Plant Biomass

Plants harvesting was carried out at the end of the experiment (after 16 weeks). Nine plants of each light level were chosen randomly and their total biomass was separated into three compartments: Leaves, stems and rhizomes, and their dry weight was calculated after drying at 70 °C (72 h).

### 3.10. Statistical Analysis

The experimental design was factorial based on Randomized complete block design (RCBD). Results are expressed as mean ± standard deviation of three replicates. Where applicable, the data were subjected to one-way analysis of variance (ANOVA) and Duncan’s Multiple Range test using the Statistical Analysis System (SAS, 1999) and Mstatc programs determined the differences between samples. *p*-value of ≤0.05 was regarded as significant.

## 4. Conclusions

This study demonstrated that different light intensities are able to change synthesis of phenolics and flavonoids components in ginger. High performance liquid chromatography analysis revealed that synthesis of flavonoids like as quercetin, catechin, epicatechin and naringenin were enhanced under low light intensity. With decreasing light intensity, the photosynthesis rate was decreased, and the result of decreasing the photosynthesis rate was a decrease in soluble carbohydrate and plant biomass in Halia Bentong and Halia Bara. According to previous studies, some flavonoid components like quercetin are able to change the rate of electron transport in photosynthesis photosystems and thereby control the photosynthesis rate. In the current study, high content of quercetin was detected in ginger with low photosynthesis rate. Contrary to flavonoids, salicylic acid is able to enhance photosynthesis rate [39] and in the current study, high content of salicylic acid was found in Halia Bara leaves grown under 790 μmol m^−2^s^−1^ with a high photosynthesis rate. Our results indicate that possible interference of flavonoids and phenolics in the photosynthetic processes may occur in the plant cell. However, with decreasing photosynthesis rate, carbons from the photosynthesis cycle shift to the shikimic acid pathway in order to produce higher flavonoid content. It could be concluded that phenolics and flavonoids are able to regulate plant growth and improve the physiological efficiency and can enhance effective partitioning of accumulate from the source-sink in plants. Further work is required to establish the phenolics and flavonoids components that may have a regulatory effect on plant growth. It would be worthwhile if future studies address this by investigating the effects of several polyphenols alone or in combination on selected plant processes.

## Figures and Tables

**Table 1.** HPLC analysis of flavonoid and phenolic compounds extracted from different parts of ginger varieties grown under different light intensities.

**Parameters**	**Halia Bentong**				**Halia Bara**			

	**790 (μmol m^−2^s^−1^)**		**310 (μmol m^−2^s^−1^)**		**790 (μmol m^−2^s^−1^)**		**310 (μmol m^−2^s^−1^)**	

	**Leaves**	**Rhizomes**	**Leaves**	**Rhizomes**	**Leaves**	**Rhizomes**	**Leaves**	**Rhizomes**
Quercetin	0.871 ± 0.031^cd^	0.803 ± 0.028^d^	0.985 ± 0.015^b^	0.902 ± 0.042^bc^	0.978 ± 0.024^b^	0.865 ± 0.027^cd^	1.123 ± 0.11^a^	0.986 ± 0.032^b^
Rutin	0.354 ± 0.0015^c^	0.311 ± 0.002^e^	0.365 ± 0.003^b^	0.451 ± 0.0045^a^	0.205 ± 0.003^b^	0.324 ± 0.002^d^	0.173 ± 0.0075^b^	0.331 ± 0.0092^d^
Epicatechin	0.092 ± 0.068^a^	0.078 ± 0.0125^a^	0.118 ± 0.014^a^	0.083 ± 0.007^a^	0.111 ± 0.017^a^	0.091 ± 0.009^a^	0.117 ± 0.004^a^	0.103 ± 0.003^a^
Catechin	0.328 ± 0.0405^e^	0.362 ± 0.021^e^	0.413 ± 0.028^d^	0.491 ± 0.019^bc^	0.455 ± 0.037^cd^	0.459 ± 0.026^cd^	0.671 ± 0.079^a^	0.533 ± 0.034^b^
Kaempferol	0.044 ± 0.012^cd^	0.045 ± 0.005^cd^	0.042 ± 0.003^d^	0.051 ± 0.004^bcd^	0.048 ± 0.004^cd^	0.061 ± 0.0045^ab^	0.053 ± 0.003^bc^	0.068 ± 0.006^a^
Naringenin	0.049 ± 0.0035^c^	0.046 ± 0.001^c^	0.094 ± 0.006^a^	0.047 ± 0.003^c^	0.039 ± 0.0045^d^	0.02 ± 0.002^f^	0.061 ± 0.0045^b^	0.028 ± 0.0035^e^
Salicylic acid	0.491 ± 0.018^d^	0.522 ± 0.041^c^	n.d	n.d	0.673 ± 0.027^a^	0.622 ± 0.055^b^	n.d	n.d
Cinnamic acid	0.124 ± 0.0087^b^	0.455 ± 0.027^a^	n.d	n.d	0.0640 ± 0.014^c^	0.059 ± 0.01^c^	n.d	n.d
Flavonoids	5.41 ± 0.44^c^	3.31 ± 0.21^d^	6.11 ± 0.326^b^	4.1 ± 0.163^d^	6.87 ± 1.21^b^	3.83 ± 0.213^d^	8.22 ± 0.514^a^	4.73 ± 0.08^c^
Phenolics	35.4 ± 1.205^a^	12.3 ± 0.41^c^	29.11 ± 2.44^b^	8.9 ± 0.31^d^	41.1 ± 1.53^a^	10.66 ± 0.44^c^	33.21 ± 1.620^b^	11.17 ± 0.33^d^

All analyses are the mean of triplicate measurements ± standard deviation; Results expressed in mg/g dry weight; Subscript letters within the same row indicate significant (*p* ≤ 0.05) differences of means within the plant materials; n.d: not detected.

**Table 2.** Alteration of flavonoids synthesis in ginger varieties by decreasing light intensity.

**Components**	**Halia Bentong**		**Halia Bara**	

	**Leaves**	**Rhizomes**	**Leaves**	**Rhizomes**
Quercetin	+13	+12	+15	+14
Rutin	+3	+45	−16	+2
Epicatechin	+28	+6	+5	+13
Catechin	+26	+36	+47	+16
Kaempferol	−5	+13	+10	+11
Naringenin	+92	+2	+56	+40
Mean	+26.1	+19	+19.5	+16

Results expressed in percent: + and −, respectively, represent increasing and decreasing.

**Table 3.** Effect of different light intensities on some physiological and morphological parameters in two varieties of ginger (*Zingiber officinale*).

**Parameters**	**Halia Bentong**				**Halia Bara**			

	**Light intensities (μmol m^−2^s^−1^)**				**Light intensities (μmol m^−2^s^−1^)**			

	**310**	**460**	**630**	**790**	**310**	**460**	**630**	**790**
Chlorophyll a	249.8 ± 2.6^b^	227.9 ± 17.8^c^	175.5 ± 20.85^d^	174.2 ± 14.4^d^	273.5 ± 3.32^a^	245.4 ± 3.4^bc^	182.5 ± 4.1^d^	184.03 ± 7.05^d^
Chlorophyll b	96.7 ± 29.7^a^	95.4 ± 25.8^a^	80.2 ± 8.29^ab^	59.06 ± 9.01^b^	103.3 ± 10.3^a^	89.1 ± 12.9^ab^	88.2 ± 19.3^ab^	94.8 ± 19.2^a^
Chlorophyll a + b	346.6 ±3 0.5^ab^	323.4 ± 12.01^b^	255.8 ± 12.6^cd^	233.35 ± 23.3^d^	376.8 ± 7.08^a^	334.5 ±9.5^b^	270.7 ± 16.7^c^	278.8 ± 26.1^c^
Chlorophyll a:b	2.7 ± 1.03^a^	2.5 ± 0.86^a^	2.2 ± 0.46^a^	2.9 ± 0.19^a^	2.6 ± 0.31^a^	2.8 ± 0.48^a^	2.1 ± 0.56^a^	1.9 ± 0.34^a^
Soluble carbohydrate	12.27 ± 0.48^g^	12.85 ± 0.37^fg^	13.75 ± 0.77^de^	17.4 ± 0.71^b^	13.47 ± 0.59^ef^	14.53 ± 0.57^d^	15.82 ± 0.49^c^	18.49 ± 0.6^a^
Photosynthesis	5.12 ± 0.992^c^	6.98 ± 0.272^b^	7.9 ± 0.738^b^	11.77 ± 0.41^a^	5.55 ± 0.533^c^	7.45 ± 0.667^b^	7.91 ± 0.757^b^	12.25 ± 1.06^a^
Stomata conductance	0.108 ± 0.031^b^	0.074 ± 0.102^b^	0.215 ± 0.04^b^	0.555 ± 0.151^a^	0.113 ± 0.014^b^	0.181 ± 0.005^b^	0.214 ± 0.021^b^	0.686 ± 0.21^a^
Transpiration	1.19 ± 0.325^b^	1.906 ± 0.545^ab^	2 ± 0.406^ab^	2.52 ± 0.522^ab^	1.978 ± 0.852^ab^	2.025 ± 0.738^ab^	1.73 ± 0.079^ab^	2.61 ± 0.36^a^
Plant biomass	47.68 ± 0.36^cd^	53.22 ± 0.61^c^	63.72 ± 2.49^b^	79.47 ± 8.92^a^	40.25 ± 6.12^d^	42.45 ± 1.81^d^	53.77 ± 3.82^c^	70.06 ± 8.41^b^

All analyses are the mean of triplicate measurements ± standard deviation; Subscript letters within the same row indicate significant (*p* ≤ 0.05) differences of means within the plant materials. Results of ch a, ch b, ch a + b expressed in μg mL^−1^; Result of soluble carbohydrate expressed in mg/g dry weight; Result of net photosynthesis expressed in μmol CO_2_ m^−2^s^−1^; Result of stomata conductance and respiration rate expressed in mmol m^−2^s^−1^; Result of dry matter is expressed in grams.

**Table 4.** Correlation between measured parameters in two varieties of Z*ingiber officinale*.

**No.**	**Characteristic**	**1**	**2**	**3**	**4**	**5**	**6**	**7**	**8**	**9**	**10**	**11**
1	Plant biomass	1										
2	Transpiration	0.5^n.s^	1									
3	Photosynthesis	0.87^**^	0.66^*^	1								
4	Stomata conductance	0.68^*^	0.69^*^	0.95^**^	1							
5	Chlorophyll a	−0.86^**^	−0.52^n.s^	0.64^*^	−0.9^**^	1						
6	Chlorophyll b	0.12^n.s^	0.22^n.s^	0.18^n.s^	0.13^n.s^	0.21^n.s^	1					
7	Chlorophyll a + b	−0.83^**^	−0.37^ns^	−0.7^*^	−0.67^*^	0.89^**^	0.43^n.s^	1				
8	Chlorophyll a:b	−0.68^*^	−0.51^n.s^	−0.86^**^	−0.79^**^	0.81^**^	−0.52^n.s^	0.48^n.s^	1			
9	Flavonoids	−0.65^*^	0.11^n.s^	−0.63^*^	−0.64^*^	0.61^*^	0.13^n.s^	0.61^*^	0.36^n.s^	1		
10	Phenolics	0.78^**^	0.6^*^	0.85^**^	0.84^**^	−0.68^*^	0.14^n.s^	−0.54^n.s^	−0.69^*^	0.03^n.s^	1	
11	Soluble carbohydrate	0.81^**^	0.65^*^	0.92^**^	0.86^**^	−0.92^**^	0.07^n.s^	−0.8^**^	−0.82^**^	−0.41^n.s^	0.82^**^	1

n.s = non significant;

* = significant at *p* ≤ 0.05;

** = significant at *p* ≤ 0.01.
